# Biogenic apatite in carbonate concretions with and without fossils investigated in situ by micro-Raman spectroscopy

**DOI:** 10.1038/s41598-023-36566-7

**Published:** 2023-06-15

**Authors:** Ryosuke Kitanaka, Motohiro Tsuboi, Yukihiro Ozaki

**Affiliations:** 1 Department of Applied Chemistry for Environment, School of Biological and Environmental Sciences, Kwansei Gakuin University, Gakuen-Uegahara 1, Sanda, Hyogo 669-1330 Japan; 2 Department of Biomedical Sciences, School of Biological and Environmental Sciences, Kwansei Gakuin University, Gakuen-Uegahara 1, Sanda, Hyogo 669-1330 Japan

**Keywords:** Geochemistry, Petrology, Analytical chemistry

## Abstract

Micro-Raman spectra of concretions with and without fossils were measured in a nondestructive manner. The band position and full width at half maximum height (FWHM) of ν_1_-PO_4_^3−^ of apatite in the concretions were analyzed to investigate the origin of apatite. The analyzed concretions were derived from the Kita-ama Formation of the Izumi Group, Japan. The micro-Raman analysis showed that the apatites in the concretions were divided into two groups: Group W (wide FWHM group) and Group N (narrow FWHM group). The apatite belonging to Group W is suggested to be biogenic apatite originating from the soft body tissues of organisms because the Sr content is high and the FWHM is similar to that of apatite in bones and teeth of present-day animals. The other apatite belonging to Group N is considered affected by the diagenetic process because of its narrow FWHM and F substitution. These features of both groups were observed regardless of the presence of fossils or absence of fossils in the concretions. This Raman spectroscopic study suggests that the apatite at the time of concretion formation belonged to Group W but was changed to Group N by the substitution of F during the diagenesis process.

## Introduction

Spherical concretions are composed of carbonates and found mainly in marine sedimentary rocks worldwide^1–3^. Concretions are well known for the presence of well-preserved ammonites and other fossils in their interior. Concretions vary in size from a few millimeters to greater than 1 m^4,5^. It has been suggested that the concretions were formed by the diffusion of organic matter^4,6,7^. However, no specific model of the concretion formation process has been reported. Yoshida et al.^5^ used tusk- shells in concretion and proposed a new model of concretion formation and its formation rate. The authors were the first to point out that the concentration gradient of elements occurs at the front of concretion and estimated the rate of concretion formation using the simple equation L = D/V (L: width of reaction front (cm); D: diffusion coefficient of HCO_3_^−^ (cm^2^/s); V: growth rate (cm/s)). The results of the estimation showed that tusk- shell concretions with a size of a few centimeters are formed as fast as several weeks to several months. This conclusion overturned the hypothesis^4^ that the concretions are formed over tens of thousands to millions of years. Additionally, Yoshida et al.^5^ showed that the δ^13^C of the soft body of the present tusk- shell and the δ^13^C of the tusk-shell concretion are consistent, indicating that the organic matter in the concretion is biogenic (originating from the soft body of the tusk- shell). It has been proposed that the concretions were formed by the rapid precipitation of bicarbonate ions (HCO_3_^−^) from decayed organisms and calcium ions (Ca^2+^) from seawater as calcium carbonate in the sediment pore after dead marine organisms were buried in the sediment^3,5^.

Studies on the major element analysis, oxygen and carbon isotope analysis, and Sr isotope ratio analysis of the concretions have been conducted^3,8–11^. However, few studies address the origin of apatite contained in concretions. In addition, the abovementioned studies were carried out by destroying and dissolving samples in acid, which requires a time-consuming process and does not provide detailed information about the molecular structure and crystallinity of the samples. In this study, we used in- situ micro-Raman spectroscopy, a nondestructive structural and analytical method. Raman spectroscopy was performed to investigate the origin of apatite. Raman spectra can reflect elemental substitutions in the crystal lattice of apatite. We focused on apatite, which is abundantly contained in concretions. If apatite is identified as biogenic, the idea of Yoshida et al.^5^ that the concretions originate from the soft parts of living organisms is reinforced.

Raman spectroscopy is vibrational spectroscopy that provides information about molecular structure and crystallinity from molecular vibration data. Since Raman spectroscopy is a nondestructive analysis method, it does not require troublesome sample preparation in most cases, and spectra can be obtained relatively simply and quickly. In addition, the ability to measure in a microscopic range (500 nm to 1 μm) makes microanalysis a specialty of this method. Because of these features, Raman spectroscopy has been used extensively in a wide range of research fields from materials sciences and biomedical sciences to earth sciences.

Apatite consists of calcium phosphate and is a major mineral that makes up animal bones and teeth^12^. The crystal structure of apatite in fossil bones and teeth has been extensively analyzed by Raman spectroscopy^13,14^. Changes in the neighboring ions in the apatite lattice slightly alter the stretching mode of ν_1_-PO_4_^3−^ (symmetric stretching vibration)^12^. Thomas et al.^13^ analyzed the position and FWHM (full width at half maximum height) of the ν_1_-PO_4_^3−^ band of geological apatites from magmatic sources and biogenic apatites from bones, teeth of present-day animals, and fossil teeth. The ν_1_-PO_4_^3−^ band position and FWHM change through the diagenetic process. The authors also analyzed the synthesized apatites (doped with CO_3_^2−^, F^−^, and Sr^2+^) and showed that the band position and FWHM of ν_1_-PO_4_^3−^ change depending on the elements present in the apatite lattice. de Sousa et al.^14^ conducted Raman analysis of apatite in fossil bones from limestone caves and showed that there are two stages of mineral composition change in bones, premortem and postmortem, and that secondary minerals are formed during the postmortem diagenetic process depending on the surrounding environment. Therefore, the chemical composition of the bone is different between pre- and postmortem, indicating that the original structure is destroyed by the diagenetic process.

It is known that the concretions have higher phosphorus content than the matrix^5,11^. However, there are no Raman spectroscopic measurements of apatite in the concretions, and thus, there is no discussion on the ν_1_-PO_4_^3−^ band position and FWHM for apatite in the concretions. Thus, we have considered that detailed analysis of apatite in the concretions by in-situ Raman spectroscopy may provide new insights into the concretion formation process. For example, it may be possible to evaluate whether the concretions are biogenic without destroying the samples.

Concretions with diameters of a few centimeters from the Kita-ama Formation of the Izumi Group in the southern part of Awaji Island, Hyogo Prefecture, Japan, were employed in this study (Supplementary Fig. 1). We investigated the apatite in concretions with fossils (sample name: UNO) and without fossils (sample name: DOS) (Supplementary Fig. 2). Not only micro-Raman studies but also quantitative analysis of the major elements and rare earth elements (REE) of the concretion (sample name: TRES) was performed (Supplementary Fig. 3). The major elements and REE of the concretion (TRES) was measured to investigate the major chemical composition of the concretions and to infer the elemental migration during the concretion formation. We analyzed the origin of the concretions from macro and micro perspectives by combining conventional destructive analysis methods and micro-Raman analysis, which has not been performed for the concretions until now.

## Results

### Raman spectra of concretions

**Figure 1:**
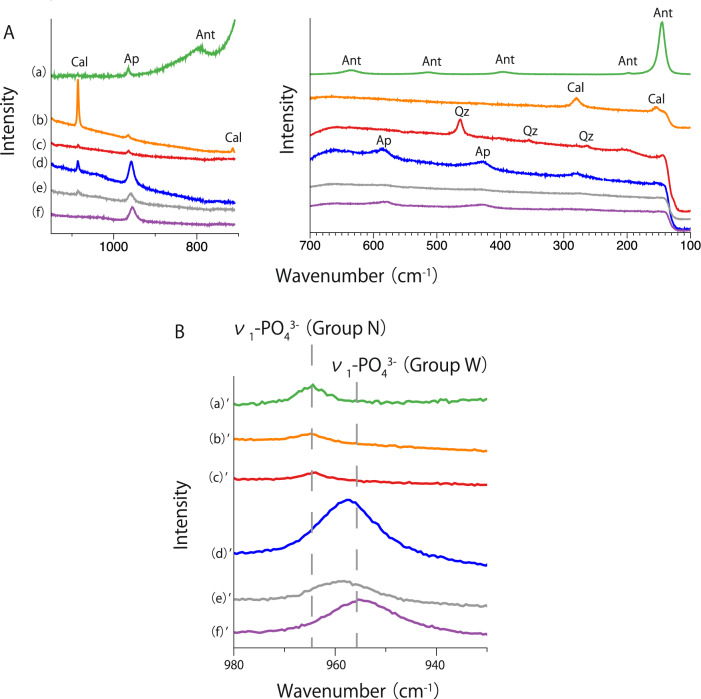
(**A**) Raman spectra in the 1150–700 cm^−1^ and 700–100 cm^−1^ regions of concretion with fossils (UNO). The ν_1_-PO_4_^3−^ band positions of phosphate (970–950 cm^−1^ region) in the top three spectra (a: green, b: orange and c: red spectra) and those in the bottom three spectra (d: blue, e: gray and f: purple spectra) are different. Abbreviations are used as follows: calcite (Cal), apatite (Ap), anatase (Ant) and quartz (Qz). (**B**) Enlarged spectra in the 980–930 cm^−1^ ν_1_-PO_4_^3−^ region. The apatite in the concretions is divided into two groups: Group N (narrow FWHM group; a′: green, b′: orange and c′: red spectra) with a higher band position and a narrow FWHM of ν_1_-PO_4_^3−^ and Group W (wide FWHM group; d′: blue, e′: gray and f′: purple spectra) with a lower band position and a wide FWHM.

Raman spectra of apatites in the 1150–700 cm^−1^ and 700–100 cm^−1^ regions of the concretion with fossils (UNO) are shown in Fig. 1A. Apatite data in the concretions are also shown in Table 1. The strongest peak due to ν_1_-PO_4_^3−^ (symmetric stretching vibration) of apatites is divided into two groups (Fig. 1B): Group N (narrow FWHM group), in which the ν_1_-PO_4_^3−^ band appears at a higher wavenumber (~ 962 cm^−1^) and the FWHM is narrower (Fig. 1B(a)′, (b)′, (c)′), and Group W (wide FWHM group), in which the ν_1_-PO_4_^3−^ band appears at a lower wavenumber (~ 954 cm^−1^) and the FWHM is wider (Fig. 1B(d)′, (e)′, (f)′). Figure 1B(a)′, (b)′, (c)′, (d)′, (e)′, and (f)′ depict enlarged spectra in the 980–930 cm^−1^ region of Fig. 1A(a), (b), (c), (d), (e), and (f), respectively. A detailed explanation of Groups N and W is given in the Discussion.

**Table 1:** ν_1_-PO_4_^3−^ band position and ν_1_-PO_4_^3−^ FWHM of the concretions with fossils (UNO) and without fossils (DOS).

Sample	Group	ν_1_-PO_4_^3−^ band position (cm^−1^)	ν_1_-PO_4_^3−^ FWHM (cm^−1^)
UNO-1	W	957.7	13.4
UNO-2	W	954.9	14.0
UNO-3	W	955.4	15.7
UNO-4	W	958.2	14.0
UNO-5	W	955.4	14.5
DOS-1	W	954.9	17.9
UNO-6	N	966.0	7.3
UNO-7	N	966.0	6.7
UNO-8	N	964.4	7.3
UNO-9	N	964.4	6.1
UNO-10	N	963.8	5.6
UNO-11	N	964.9	7.3
UNO-12	N	964.9	7.8
UNO-13	N	964.9	7.8
UNO-14	N	964.9	8.4
UNO-15	N	963.8	9.5
UNO-16	N	962.1	8.9
UNO-17	N	964.4	6.1
UNO-18	N	962.1	10.6
UNO-19	N	964.9	6.7
UNO-20	N	964.4	6.7
DOS-2	N	963.3	10.6
DOS-3	N	964.4	5.6
DOS-4	N	964.9	6.1
DOS-5	N	965.5	6.7
DOS-6	N	964.9	8.4
DOS-7	N	964.9	7.3
DOS-8	N	964.4	5.6
DOS-9	N	964.4	7.3

Other apatite peaks are observed at 585 cm^−1^ (ν_4_: bending) and 425 cm^−1^ (ν_2_: bending)^15^ (Fig. 1A(d) and (f)). In the area where apatite is present, peaks due to calcite are observed at 1085 cm^−1^ (ν_1_: symmetric stretching vibration), 712 cm^−1^ (ν_4_: symmetric deformation), 278 cm^−1^ (translational lattice mode), and 154 cm^−1^ (translational lattice mode)^16^. In some areas, anatase peaks were observed at 636, 515, 395, 198, and 144 cm^−1^ (Fig. 1A(a))^17^ and quartz peaks were observed at 463, 355 and 263 cm^−1^ (Fig. 1A(c))^18^. Raman spectra of apatites in the concretion without fossils (DOS) were also measured, and the obtained spectra showed characteristics similar to those of the concretion with fossils (UNO). Raman analysis was also carried out for the matrix. In the Raman spectra of the matrix, peaks assigned to quartz and anatase were mainly observed (Supplementary Figs. 4, 5). A portion of calcite was also observed in the spectra (Supplementary Fig. 6). The Raman spectra of the matrix were compared with the RRUFF database (http://rruf.info/^19^), and the observed results roughly correspond to the spectra in the literature (Supplementary Figs. 4–6).

### Phosphorus mapping by WDXRF

**Figure 2:**
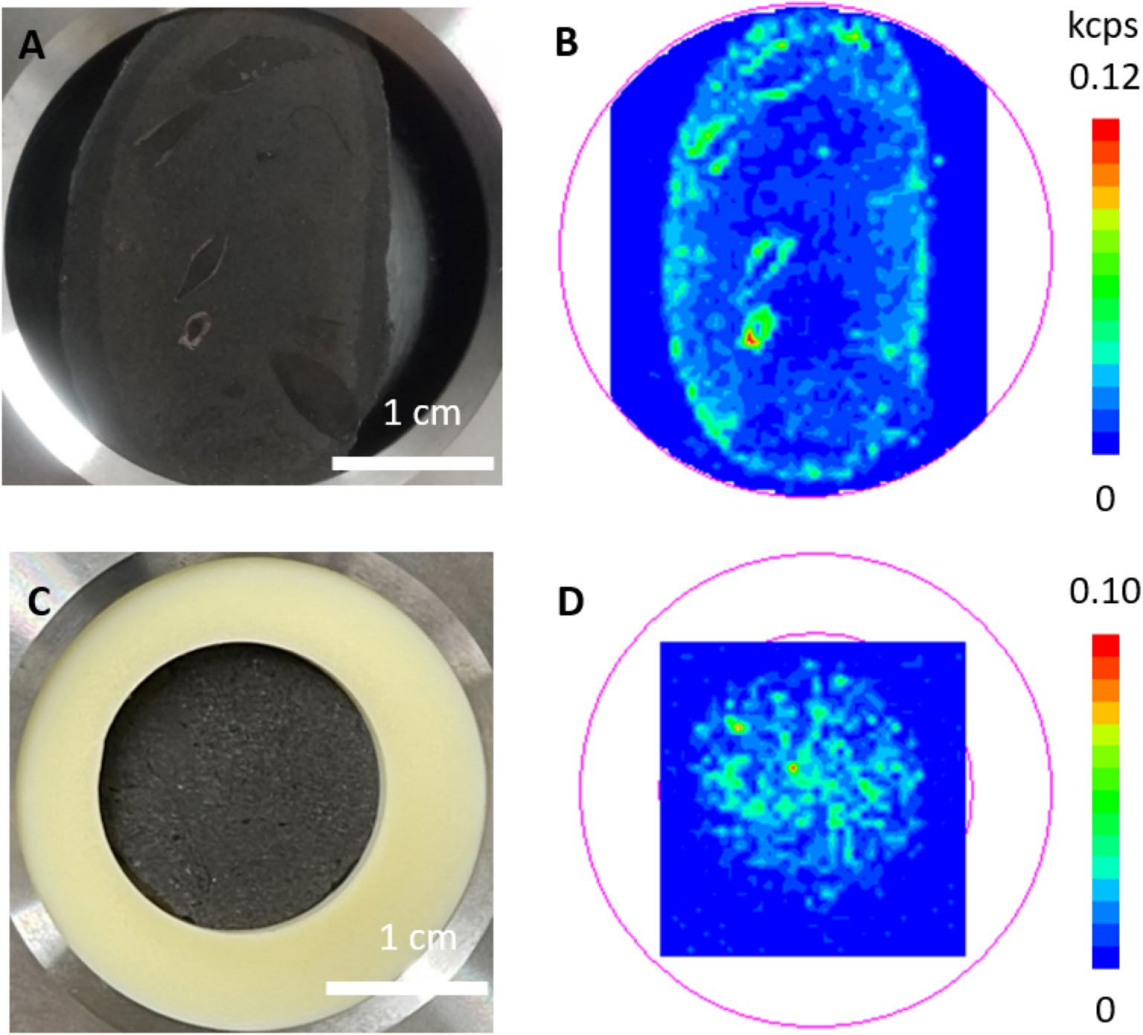
(**A**) Cross- section of the concretion with fossils (UNO). (**B**) XRF P X-ray intensity in the concretion. Phosphorus is especially concentrated in the fossils. (**C**) Cross-section of the concretion without fossils (DOS). The concretion was too small to fit the holder, and thus, it was adjusted with the Teflon plate. (**D**) XRF P X-ray intensity in the concretion. Phosphorus is widely distributed.

Phosphorus mapping was developed using wavelength dispersive X-ray fluorescence (WDXRF) spectrometer for concretions with (UNO) and without fossils (DOS). The results are displayed in Fig. 2, which shows that the concretion with fossils (UNO) has a high phosphorus content at the fossils (Fig. 2B), while the concretion without fossils (DOS) has a phosphorus content all around (Fig. 2D).

### Major and rare earth element (REE) composition of concretion

**Figure 3:**
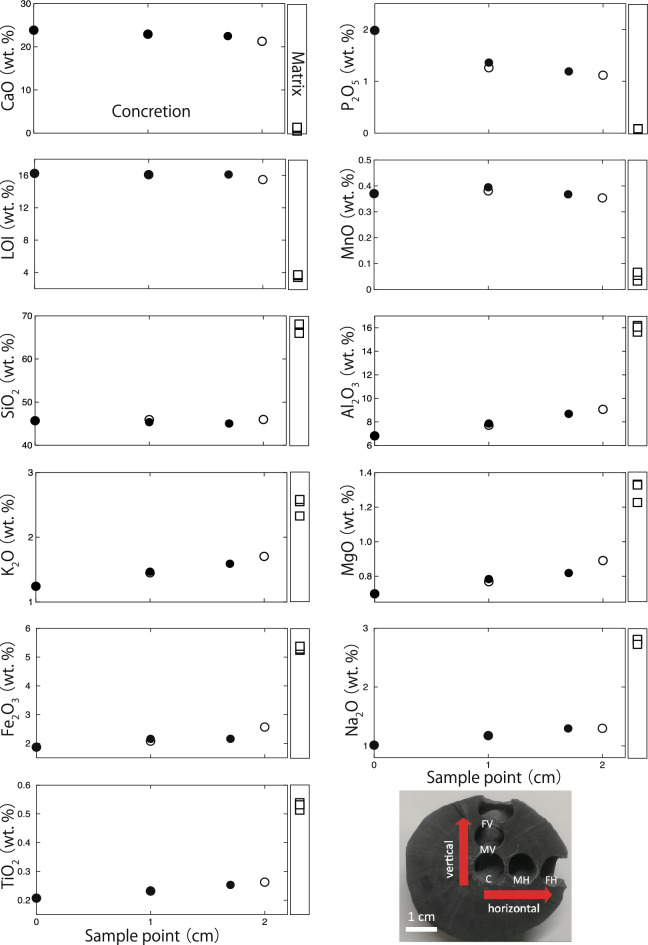
Major elements in the concretion (TRES). The horizontal axis indicates the sampling point from 0 to 2.0 cm. White circles (〇) show horizontal sampling points, and black circles (●) show vertical sampling points. The rectangle on the right in each figure represents the matrix. The data measured by WDXRF were unified to a total of 100%. Abbreviations are used as follows: core (C), mantle-horizontal (MH), front-horizontal (FH), mantle-vertical (MV) and front-vertical (FV).

The major elements (Si, Ti, Al, Fe, Mn, Mg, Ca, Na, K and P) of the concretion (TRES) and matrix from the Kita-ama formation were measured by WDXRF. The results are presented in Fig. 3 and Supplementary Table 1. The elemental trends did not change when the presented concretions were horizontally or vertically measured. Therefore, the concretions were considered to grow in concentric circles.

**Figure 4:**
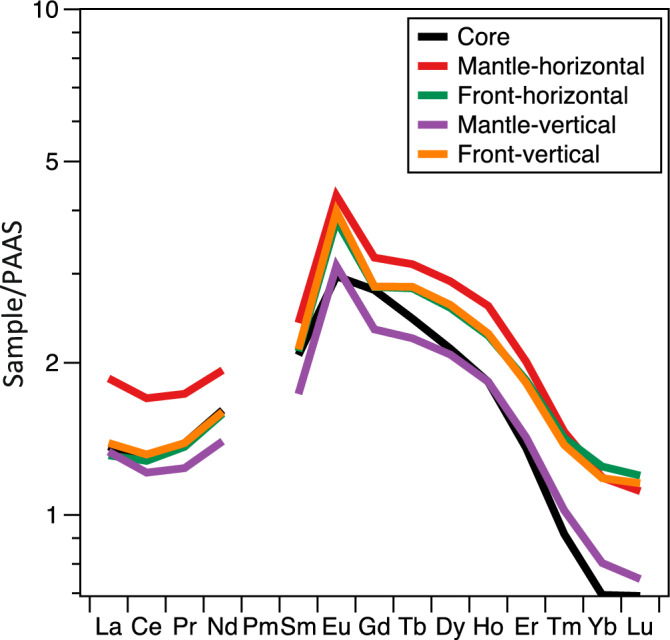
REE pattern of concretion (TRES). The REE data are normalized to Post Archean average Australian sedimentary rocks (PAAS)^20^.

The 15 REE (Y, La, Ce, Pr, Nd, Sm, Eu, Gd, Tb, Dy, Ho, Er, Tm, Yb and Lu) in the concretion (TRES) were determined using ICP-MS (Supplementary Table 2). The REE pattern normalized by PAAS^20^ is shown in Fig. 4; the REE pattern is a bell-shaped pattern enriched in MREE, which is similar in the core, mantle, and front. A significant positive Eu anomaly and a slight negative Ce anomaly (Ce/Ce* = 0.93–0.96) are also shown.

## Discussion

**Figure 5:**
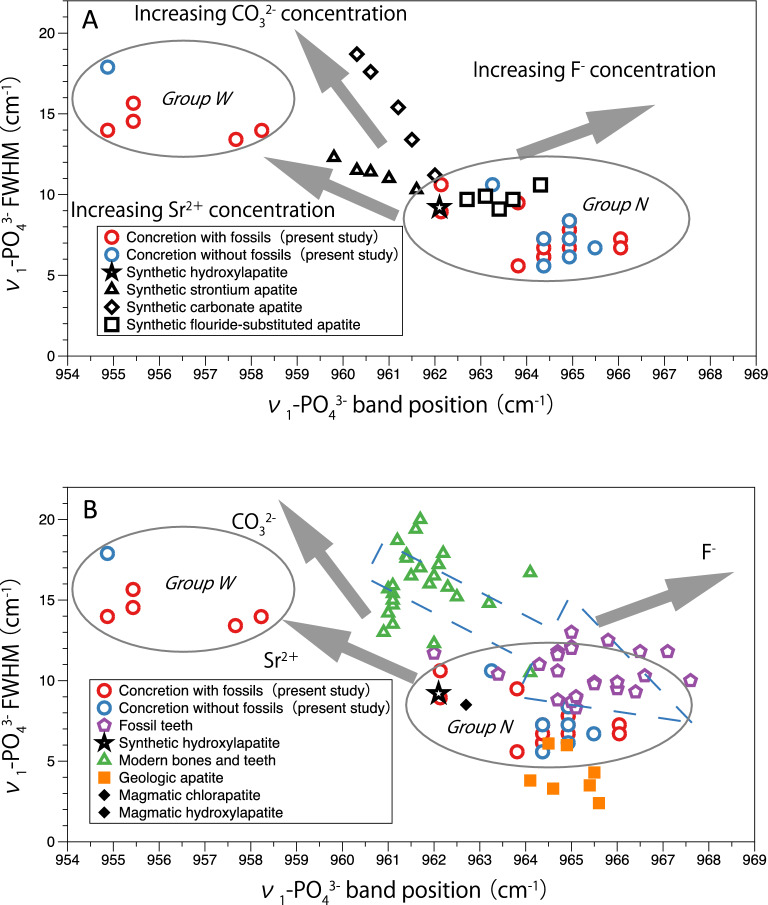
(**A**) ν_1_-PO_4_^3−^ band position (cm^−1^) versus ν_1_-PO_4_^3−^ FWHM (cm^−1^) of synthetic apatite samples and concretion samples (present study). The synthetic apatite sample data are obtained from Thomas et al.^13^. The gray arrows indicate the amount of each element in the apatites. (**B**) ν_1_-PO_4_^3−^ band position (cm^−1^) versus ν_1_-PO_4_^3−^ FWHM (cm^−1^) of teeth samples from fossil, modern animal bones and teeth, geologic apatites and concretion samples (present study). Magmatic hydroxylapatite and chlorapatite had the same ν_1_-PO_4_^3−^ band position and FWHM. The apatite sample data are obtained from Thomas et al.^13^, with the exception of the concretion samples. The blue dotted arrow indicates the direction of progression of the diagenetic process. The gray arrows indicate the amount of each element in apatites.

Thomas et al.^13^ investigated the position and FWHM of the ν_1_-PO_4_^3−^ band of geological apatites from magmatic sources and biogenic apatites, such as those from bones and teeth of present-day animals and fossil teeth. Figure 5A depicts the ν_1_-PO_4_^3−^ FWHM vs. its position for synthetic apatites examined by Thomas et al.^13^ and the concretions with and without fossils (present study). Additionally, Fig. 5B depicts the ν_1_-PO_4_^3−^ FWHM vs. its position for geological apatites from magmatic sources and biogenic apatites such as those from bones and teeth of present-day animals, fossil teeth investigated by Thomas et al.^13^ and concretions with and without fossils (present study). The authors demonstrated that the ν_1_-PO_4_^3−^ band position and FWHM of fossil teeth significantly change during the diagenesis process (Fig. 5B). For example, the authors pointed out that biogenic apatites, such as those of animal bones and teeth, showed the ν_1_-PO_4_^3−^ band position at a lower wavenumber and had a wider FWHM (Fig. 5B). On the other hand, geological apatites, such as those in igneous rocks, had the ν_1_-PO_4_^3−^ band position at a higher wavenumber and a narrower FWHM (Fig. 5B). It is also known that the band position of ν_1_-PO_4_^3−^ becomes higher after the diagenesis process and that the FWHM becomes narrower after the diagenesis process^13^. The blue dotted arrow indicates the direction of progression of the diagenetic process (Fig. 5B)^13^. Therefore, the differences in the band position and FWHM of ν_1_-PO_4_^3−^ of apatite provide information about the origin of apatite and the influence of its diagenetic process^13^.

In this study, we focused on the band position and FWHM of ν_1_-PO_4_^3−^ following the study by Thomas et al.^13^. The data of this study are plotted with those of Thomas et al.^13^, as shown in Fig. 5. Although Thomas et al.^13^ and de Sousa et al.^14^ investigated the relationship between the band position and the FWHM of ν_1_-PO_4_^3−^ apatites for fossil teeth and bones, this is the first time that apatite in concretions has ever been analyzed. The plots in Fig. 5A also show that the appearances in the concretions are divided into two groups: Group W (wide FWHM group), in which the ν_1_-PO_4_^3−^ band appears at a lower wavenumber and the FWHM is wider, and Group N (narrow FWHM group), in which the ν_1_-PO_4_^3−^ band position appears at a higher wavenumber and the FWHM is narrower. The gray arrows indicate the amount of each element in the apatites. Group W is enriched in Sr and has a similar FWHM to those of apatites in bones and teeth of present-day animals. In contrast, Group N is enriched in F and plots between geologic apatite and fossil teeth. These features are observed regardless of the presence or absence of fossils in the concretions. It is unclear whether the phosphorus is of soft part origin of organisms or not. However, the high values of FWHM in the Group W apatite indicates that it is definitely of biogenic origin.

Sr is coprecipitated and incorporated into concretions when carbonates are precipitated because its ionic radius is close to that of Ca^21^. Since the crystal structure of apatite is highly diverse, Sr is substituted within the apatite crystal lattice. The FWHM represents the crystallinity of the mineral. At the time of concretion formation, Sr substitution in the apatite crystal lattice is considered to have caused the apatite crystals to become inhomogeneous, resulting in broad peaks. The nonobservation of apatite in the matrix and the observation of apatite belonging to Group W outside the fossil in the concretion suggest that the apatite of Group W originates from the soft body of the organisms. This Raman spectroscopic study is the first to reach this conclusion and reinforces the idea of Yoshida et al.^5^ that the concretions were formed by the corrosion of the soft body of organisms. Therefore, apatite belonging to Group W is considered the apatite at the time of concretion formation that has not been affected by diagenetic processes and that is derived from the soft parts of organisms.

In contrast, Group N plots between geologic apatite and fossil teeth (Fig. 5B). With the diagenetic process, the band position of ν_1_-PO_4_^3−^ shifts toward a higher wavenumber, and the FWHM becomes narrower^13,14^. Thomas et al.^13^ defined the ν_1_-PO_4_^3−^ band position of apatite in fossils undergoing diagenetic processes as above 965 cm^−1^ and its FWHM as below 13 cm. Therefore, the apatite in the concretions belonging to Group N is considered affected by diagenetic processes. This Raman spectroscopic study suggests that the apatite at the time of concretion formation belongs to Group W but was changed to Group N by the substitution of F during the diagenesis process (the blue dotted arrows in Fig. 5B). Yoshida et al.^5^ assumed that the tusk- shells contained in the concretions were aragonite, and thus, the concretions preserved the fossils in the state when they were formed. However, this study suggests that the concretions have undergone diagenetic processes to the extent that the apatite has undergone alteration.

The result of phosphorus mapping using WDXRF indicates that the phosphorus content is high at the fossils in the concretion with fossils (UNO) (Fig. 2B). Since we do not know the identity of the fossils in the concretion, there are two possible explanations: either the phosphorus was originally present in the site or it was concentrated in the fossil portions of the concretion. We consider the former possibility. Animal bones and teeth are mainly composed of apatite, which exists as hydroxyapatite (Ca_10_(PO_4_)_6_(OH)_2_). Therefore, if the fossils are derived from animal bones or teeth, the apatite in the concretions is mainly derived from them. Raman measurements of the fossils in the concretion (UNO) showed both Group W apatite with a wider FWHM and Group N apatite with a narrower FWHM (Fig. 1B). Since the fossils in the concretions are known to remain in a state close to that at the time of formation^5^, it is not surprising that both Group W in its prenatal state and Group N in an advanced state of fossilization were observed. The latter possibility of phosphorus enrichment in the fossils cannot be rejected. Therefore, there are two different ideas about the behavior of phosphorus in this study, and we cannot choose just one. As in Yoshida et al.^5^, analysis and comparison of tusk- shells from present-day and tusk-shell concretions may lead to a better understanding of the phosphorus concentration process.

WDXRF measurements of the concretion (TRES) indicate that they contain more CaO, P_2_O_5_, MnO, and LOI than the matrix (Fig. 3). The above results are similar to those obtained by Muramiya et al.^11^ and Yoshida et al.^22^. However, in addition to the above elements, MgO and Fe_2_O_3_ were dominant in the concretions^11,22^. This finding suggests that the basic formation process is the same, such as the concentration of CaO, P_2_O_5_, and MnO in the concretions, however, some of the elements concentrated in the concretions are different depending on the geological environment in which the concretions were formed. Phosphorus (apatite), which was the focus of this study, is high in the center of the concretion and decreases toward the outside (Fig. 3). The major element analysis of the giant concretion analyzed by Muramiya et al.^11^ also indicates that P_2_O_5_ is the highest at the center of the concretion. The concretion (TRES), which contains fossils in its center, suggests that the phosphorus may have originated from its soft body parts.

In this study, concretions (carbonates + sediments) were leached with acetic acid, and REE were measured only in the carbonate part of the concretion. Figure 4 shows that the REE in the concretion are more abundant than those in the PAAS. Additionally, the shape is MREE-rich. The REE content of the concretion is higher than that of PAAS (we assume that PAAS is the matrix), and the REE pattern is not flat, suggesting that the matrix is not the only source of REE in the concretion. There are three possible sources of REE in the concretion: the surrounding matrix, seawater, and sediments in the concretion. It is known that concretions are formed in a short period (several weeks to several months) and are highly resistant to weathering^5^. Therefore, the concretion is considered to retain its former condition once it is formed. Additionally, the concretions have low porosity^11^, which makes them less permeable and subject to external influences. Therefore, it is suggested that REE may have been supplied by sediments in the concretion, considering the enclosed environment of the concretion. Future analysis and comparison of the REE of the sediments in the concretion and the surrounding matrix may be helpful in evaluating the contribution of the REE of the sediments in the concretion.

Additionally, Fig. 4 shows that the REE pattern of the concretion (TRES) is MREE-rich. This MREE-rich pattern is similar to the REE pattern of the phosphate concretions analyzed by Herbert & Compton^23^. This MREE-rich pattern may be attributed to the translocation of MREE-rich fecal pellets into the sediment, which were incorporated into the phosphate during initial burial diagenesis^24^. Although the concretions in this study are calcite, phosphorus may have affected REE concentrations. Matýsek and Jirásek^25^ measured REE in rhodochrosite and phosphate concretions and discovered that ΣREE (total REE) correlated with phosphorus content. Therefore, apatite may also be associated with REE enrichment in concretions. The significant positive Eu anomaly also suggested precipitation in a reductive (anoxic) environment^26^.

**Figure 6:**
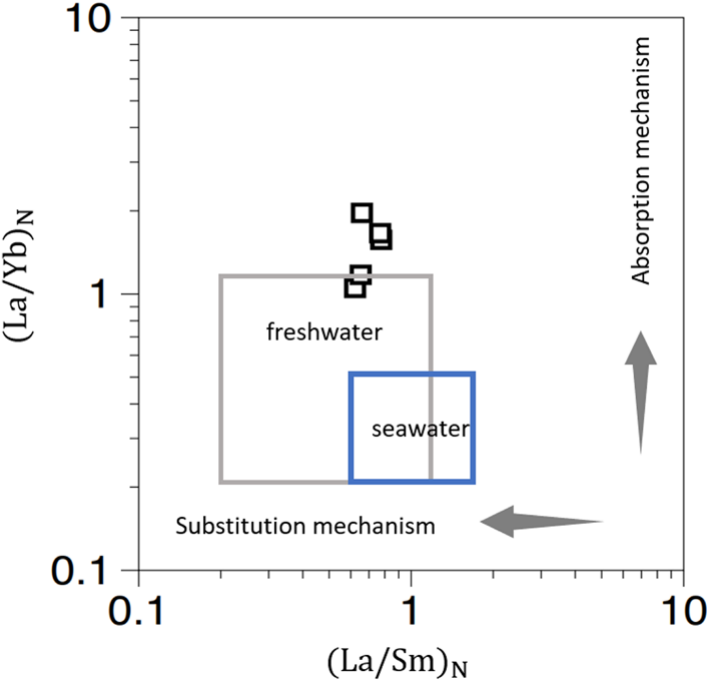
$\left( {{\text{La}}/{\text{Yb}}} \right)_{{\text{N}}}$ versus $\left( {{\text{La}}/{\text{Sm}}} \right)_{{\text{N}}}$ ratios in the concretion (TRES) and fresh and seawater^27,28^. N index indicates normalization to PAAS^20^.

From $\left( {{\text{La}}/{\text{Yb}}} \right)_{{\text{N}}}$ and $\left( {{\text{La}}/{\text{Sm}}} \right)_{{\text{N}}}$, the influence of the diagenetic process of the concretions was evaluated (Fig. 6). From previous studies, the REE in fossil bones have been measured, and the diagenetic process has been evaluated using $\left( {{\text{La}}/{\text{Yb}}} \right)_{{\text{N}}}$ and $\left( {{\text{La}}/{\text{Sm}}} \right)_{{\text{N}}}$^27–29^. $\left( {{\text{La}}/{\text{Yb}}} \right)_{{\text{N}}}$ is hardly affected by the substitution mechanism but is affected by the adsorption mechanism. Conversely, $\left( {{\text{La}}/{\text{Sm}}} \right)_{{\text{N}}}$ is hardly affected by the adsorption mechanism but is affected by the substitution mechanism^28^. The $\left( {{\text{La}}/{\text{Yb}}} \right)_{{\text{N}}}$ and $\left( {{\text{La}}/{\text{Sm}}} \right)_{{\text{N}}}$ of seawater are 0.2–0.5 and 0.6–1.6, respectively. On the other hand, the $\left( {{\text{La}}/{\text{Yb}}} \right)_{{\text{N}}}$ and $\left( {{\text{La}}/{\text{Sm}}} \right)_{{\text{N}}}$ of freshwater (rivers and lakes) has a wide range of 0.2–1.2^28^. The $\left( {{\text{La}}/{\text{Yb}}} \right)_{{\text{N}}}$ and $\left( {{\text{La}}/{\text{Sm}}} \right)_{{\text{N}}}$ of concretion (TRES) fall between 1.0–2.0 and 0.6–0.8, respectively. Samples with $\left( {{\text{La}}/{\text{Sm}}} \right)_{{\text{N}}}$ < 0.3 were used to evaluate the influence of late diagenetic processes, and their REE patterns were described as bell-shaped^27^. The $\left( {{\text{La}}/{\text{Sm}}} \right)_{{\text{N}}}$ > 0.3 in the present concretion suggests that it is not affected by the late diagenetic process and has not undergone recrystallization. However, the REE pattern in this study is bell-shaped and does not always agree with that of Kiseleva et al.^27^. The relation between $\left( {{\text{La}}/{\text{Yb}}} \right)_{{\text{N}}}$ and $\left( {{\text{La}}/{\text{Sm}}} \right)_{{\text{N}}}$ (Fig. 6) suggests that the concretions are no as affected by the diagenetic process, but the Raman spectroscopic analysis (Fig. 1B) shows that the apatite is divided into Groups N and W. The apatite belonging to Group N, which was affected by diagenetic processes, was more frequently observed. Therefore, it was considered that the apatite had undergone a diagenetic process at least to the extent of alteration of apatite. This process may have influenced the above results. Additionally, REE in the fossil bones are mainly contained in apatite, while in the concretions they are contained not only in apatite but also in calcite, which may account for the difference.

## Conclusion

In this study, apatites in two types of concretions (with and without fossils) were explored using micro-Raman spectroscopy. Based on the ν_1_-PO_4_^3−^ band position and FWHM of apatite in the concretions, it was determined that there are two types of apatites in the investigated concretions: Group W and Group N. The apatite belonging to Group W is suggested to be biogenic apatite originating from the soft body of organisms because the Sr content is high and the FWHM is similar to that of biogenic apatite, such as modern animal teeth and bones. The other apatite belonging to Group N is considered affected by the diagenetic process because of its narrow FWHM and F substitution. Group N has characteristics of geologic apatite. Therefore, it is likely that the apatite at the time of the concretion formation was in Group W, but as the concretions underwent diagenetic processes, F was substituted, and the apatite changed to Group N. The band position and FWHM of ν_1_-PO_4_^3−^ were similar to each other regardless of the presence or absence of fossils in the concretions. In addition, the similar trends in the band position and FWHM of ν_1_-PO_4_^3−^ suggest that the apatite in the concretions without fossils also arises from the soft-body parts of the organisms. Therefore, it is considered that the concretions that do not contain fossils were formed through the same formation process as those that contain fossils. The results of phosphorus mapping by WDXRF indicate that the phosphorus content is high in the fossils of the concretion. In addition to the nondestructive analysis method, the major elements were analyzed using conventional destructive analysis methods. It was revealed that P_2_O_5_ was the highest in the core of the concretion, that the basic formation processes of CaO, P_2_O_5_, and MnO concentrations in concretion are the same, and that the elements concentrated in concretion differ depending on the geological environment. The results of REE measurements suggest that the concretion was not strongly affected by the diagenetic process, whereas the Raman analysis results indicate that the concretion was altered to the extent that apatite was altered. This study reinforces the idea of Yoshida et al.^5^ that concretions originate from the soft parts of organisms and demonstrates that micro-Raman spectroscopy, a nondestructive analytical method, is powerful for investigating concretions. In recent years, Raman imaging methods have been developed and used in various research fields. The Raman imaging technique will be a powerful tool and provide a novel information such as detailed distribution of apatite, and spatial repartition of Group N and Group W inside concretions in the future study.

## Methods

In this study, concretions (diameter is a few cm) with fossils (sample name: UNO) and without fossils (sample name: DOS) were collected in the southern part of Awaji Island, Hyogo Prefecture, Japan (Supplementary Figs. 1, 2). We obtained the concretions from the Kita-ama Formation. The sampling point is located north of the Median Tectonic Line in the Izumi Group, which was deposited in the Upper Cretaceous period^30^. The Izumi Group unconformably overlies the Upper Cretaceous Ryoke volcano-plutonic rock series (ca. 100–80 Ma) and is distributed from the Izumi Mountains to Matsuyama City in Ehime Prefecture, Shikoku, extending 300 km east to the west with a thickness greater than 10,000 m^30,31^. The Izumi Group was rapidly deposited by turbidites and consists mainly of sandstone and mudstone layers^32^. The Izumi Group is famous for generating a variety of fossils, such as ammonites, and geological studies are being actively conducted^30,32,33^.

The concretions were cut in half with a rock cutter; one half was used for micro-Raman spectra analysis and the other half was used for phosphorus mapping by wavelength dispersive X-ray fluorescence (WDXRF) spectrometer. We also collected samples from several parts (core, mantle and front) of the concretion (sample name: TRES) and measured their major elements and REE. The concretion (TRES) has a fossil, and a layered pattern is visible (Supplementary Fig. 3).

### Micro-Raman spectra measurements

Thin sections of concretions (UNO and DOS) and matrix were prepared, and their Raman spectra were measured using a micro-Raman spectrometer (micro-Raman spectrometer; Raman-750, Seishin Syoji). The Raman spectra were collected with an excitation wavelength of 532 nm (50 mW) using a 100 × /0.8NA objective lens (OLYMPUS) and 1/10 attenuation filter. The irradiation time ranged from 4 to 30 s, the cumulative number ranged from 1 to 64 times, the diffraction grating was 1800, the spatial resolution was 1 μm, the CCD detector pixels were 1024 × 256 elements, the laser spot diameter was 1 μm with a 100 × objective lens, and the pinhole diameter was 200 μm. The wavenumber resolution of the Raman spectrometer was 1.8 cm^−1^. The positions of the bands were determined from peak tops, and the FWHMs were measured after subtracting the spectral backgrounds.

### Mapping of phosphorus in the concretions

Phosphorus in the concretions (UNO and DOS) was mapped by a wavelength dispersive X-ray fluorescence (WDXRF; XRF-1800, Shimadzu Corporation) spectrometer. Concretion surfaces polished with diamond powder were used as the measurement samples. The measurement conditions are listed as follows: irradiation time of 1 s, analysis diameter of 0.5 mm, no BG correction, and acceleration voltage and current for the X-ray tube of 40 kV and 95 mA, respectively.

### Major and rare earth elements (REE) in the concretion

Major elements (Si, Ti, Al, Fe, Mn, Mg, Ca, Na, K and P) and REE (Y, La, Ce, Pr, Nd, Sm, Eu, Gd, Tb, Dy, Ho, Er, Tm, Yb and Lu) were measured for the concretion (TRES) collected from the Kita-ama Formation. Major elements and REE were measured by WDXRF and inductively coupled plasma mass‒spectrometry (ICP-MS; 7500 series ICP-MS, Agilent Technologies), respectively. The concretion was drilled, and several parts (core, mantle and front) were subjected to the measurement of major elements and REE.

Powder samples of drilled concretion were heated at 500 °C for 2 h and subsequently heated at 1000 °C for 3 h. The decrease in sample mass was measured, and the LOI (loss on ignition) was calculated. A glass bead was prepared from the sample after measuring the LOI. The flux was lithium tetraborate, and the sample to flux mixing ratio was 0.7:6 g. The X-ray tube target was Rh. The accelerating voltage and current for the X-ray tube were 40 kV and 70 mA, respectively.

Fifteen REE (Y, La, Ce, Pr, Nd, Sm, Eu, Gd, Tb, Dy, Ho, Er, Tm, Yb and Lu) were measured. Two milliliters of acetic acid was added to the concretion powder, and the powder was allowed to stand for 5 h at room temperature. Then, the concretion was centrifuged at 3500 rpm for 15 min to separate the carbonate and sediment portions. The upper portion (carbonate) was collected and isolated with cation exchange resin ($DOWEX^{TM}$ 50 W × 8 200–400 Mesh (H) Cation exchange resin). The REE fraction was dissolved in nitric acid for analysis by ICP-MS.

## Supplementary Information

Supplementary Information.

## Data Availability

All Raman spectra data used during this study are available from the corresponding author on reasonable request.
