# Supplementary material for: Biogenic apatite in carbonate concretions with and without fossils investigated in situ by micro-Raman spectroscopy
Source: Sci Rep. 2023 Jun 15;13:9714. doi: 10.1038/s41598-023-36566-7 (PMC10272169; doi:10.1038/s41598-023-36566-7)
Supplement: Supplementary file 1 — Supplementary Information. [file 41598_2023_36566_MOESM1_ESM.pdf]

	Core	Mantle-h	Front-h	Mantle-v	Front-v	RK β	RK γ	red3
	Concretion					Matrix		
Fe_2O_3	2.18	2.43	2.97	2.51	2.50	5.09	5.06	5.14
MnO	0.43	0.45	0.41	0.46	0.43	0.03	0.05	0.06
TiO_2	0.24	0.27	0.30	0.27	0.29	0.58	0.60	0.59
CaO	27.73	26.79	24.57	26.82	26.00	0.69	1.33	1.59
K_2O	1.45	1.70	1.97	1.71	1.84	2.79	3.04	3.08
P_2O_5	2.31	1.48	1.29	1.58	1.38	0.09	0.11	0.10
SiO_2	53.12	53.73	53.10	52.79	52.09	71.77	68.93	68.47
Al_2O_3	7.92	9.02	10.47	9.15	10.04	16.47	16.85	16.67
MgO	0.81	0.90	1.03	0.91	0.95	1.25	1.34	1.33
Na_2O	1.18	1.37	1.50	1.38	1.50	2.76	2.74	2.65
LOI	18.89	18.81	17.89	18.80	18.63	3.70	3.89	3.99
Total	116.24	116.95	115.50	116.38	115.63	105.22	103.93	103.67

Supplementary Table 1. Major element composition of the concretion (TRES) and matrix, given in wt. %. Major elements were analyzed by WD-XRF. h: horizontal, v: vertical.

Sample	Core	Mantle-h	Front-h	Mantle-v	Front-v
Y	70.4	96.8	77.2	68.4	80.1
La	60.9	83.0	58.4	59.4	61.8
Ce	113.5	150.1	112.9	107.0	116.2
Pr	14.1	17.6	13.8	12.6	14.1
Nd	60.1	72.1	59.2	52.2	59.7
Sm	14.3	16.5	14.5	11.9	14.6
Eu	3.6	5.2	4.6	3.8	4.8
Gd	16.8	19.5	17.1	14.1	17.1
Tb	2.2	2.8	2.5	2.0	2.5
Dy	11.4	15.4	13.7	11.1	13.9
Ho	1.9	2.7	2.4	1.9	2.4
Er	4.2	6.2	5.7	4.4	5.6
Tm	0.4	0.7	0.6	0.5	0.6
Yb	2.1	3.6	3.8	2.4	3.6
Lu	0.3	0.5	0.5	0.3	0.5
ΣREE(La-Lu)	305.7	395.9	309.7	283.5	317.4

Supplementary Table 2. REE composition of concretion (TRES), given in ppm. REE were analyzed by ICP–MS. h: horizontal, v: vertical.

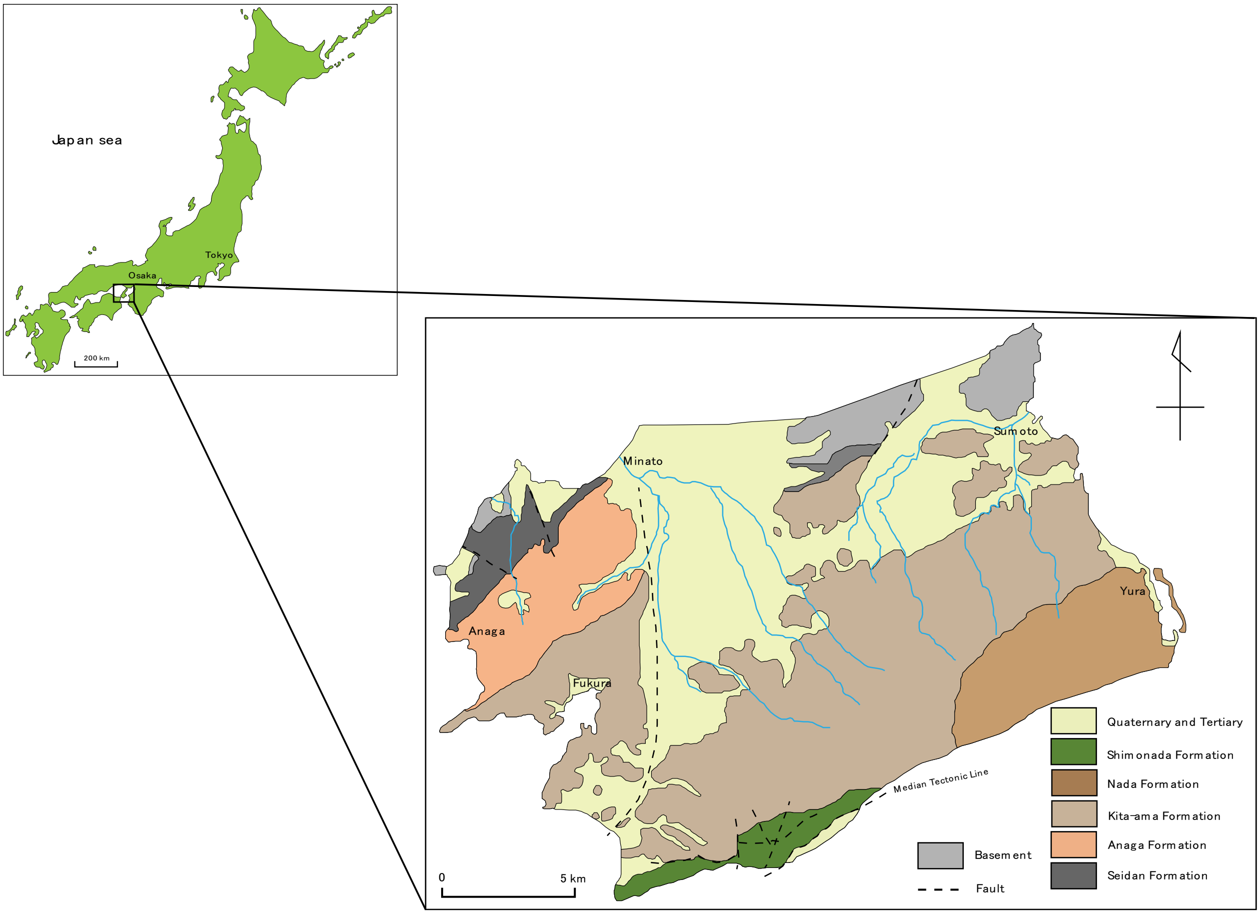

Supplementary Figure 1. Geologic map of the southern part of Awaji Island, Hyogo Prefecture, Japan, showing the distribution of the Izumi Group (modified from Morozumi [32] and, Terada and Handa[33]). Samples were collected from the Kita-ama Formation.

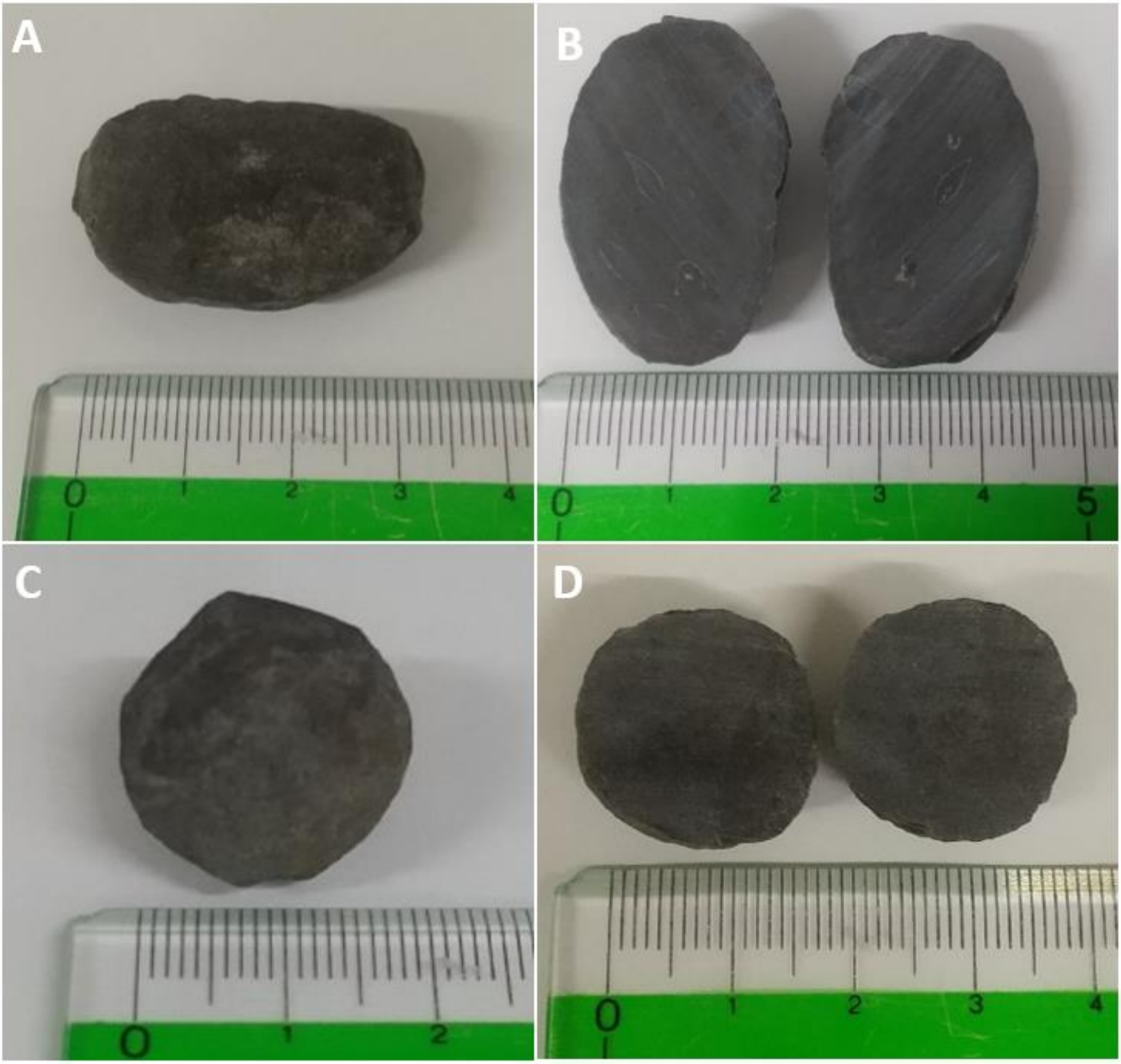

Supplementary Figure 2. (A) Concretion containing fossils (sample name; UNO). Shape: elliptical, long side: 3.2 cm, short side: 2.1 cm, and thickness: 1.8 cm. (B) Cross- section of concretion (UNO). (C) Concretion not- containing fossils (sample name; DOS); it is almost spherical, and its diameter is 2 cm. (D) Cross- section of concretion (DOS).

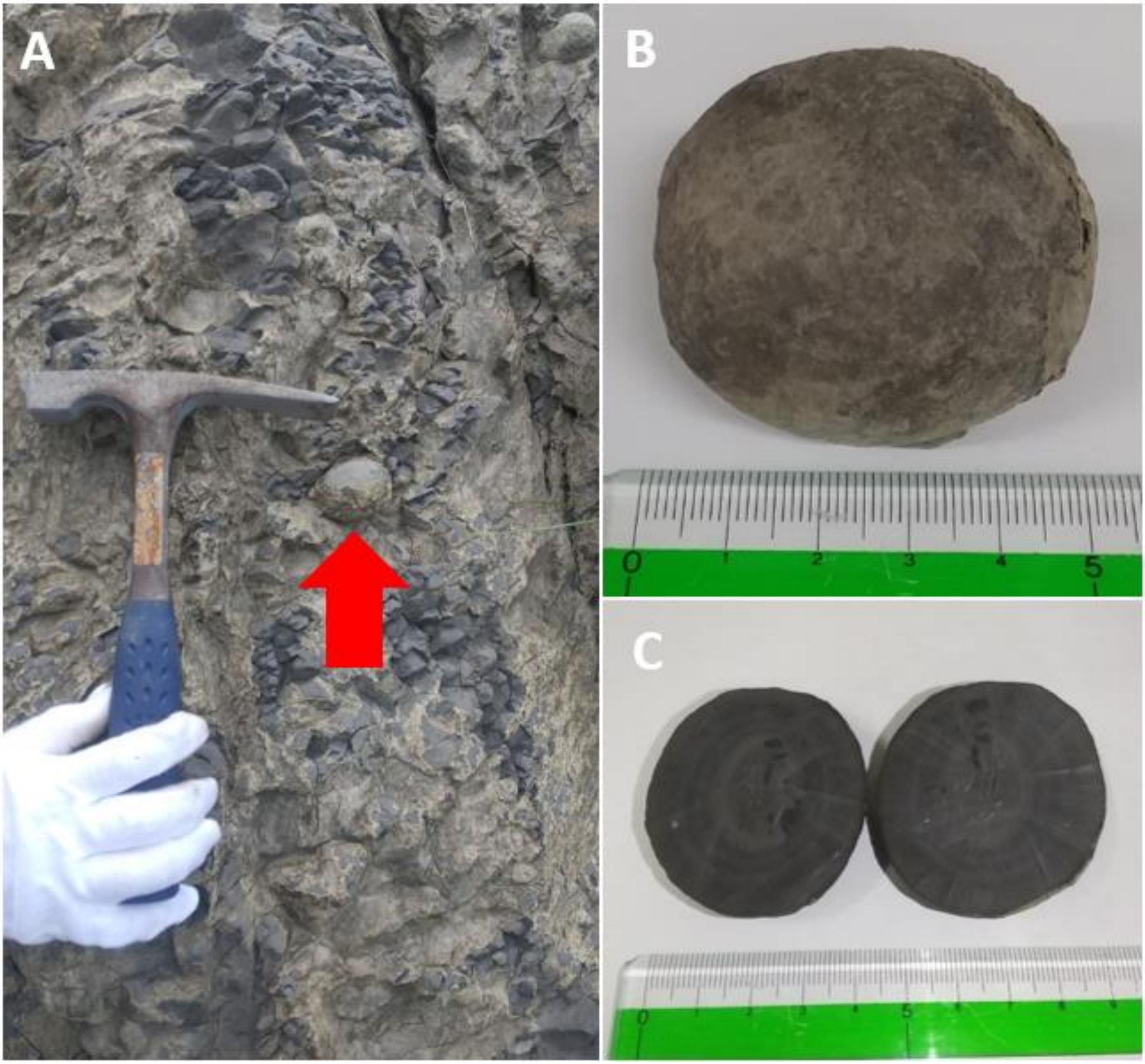

Supplementary Figure 3. (A) Concretion (sample name; TRES). (B) Shape: almost spherical, the long side: 4.9 cm, the short side: 4.6 cm, thickness: 3.7 cm. (C) The concretion contains fossils, and black crystals are observed.

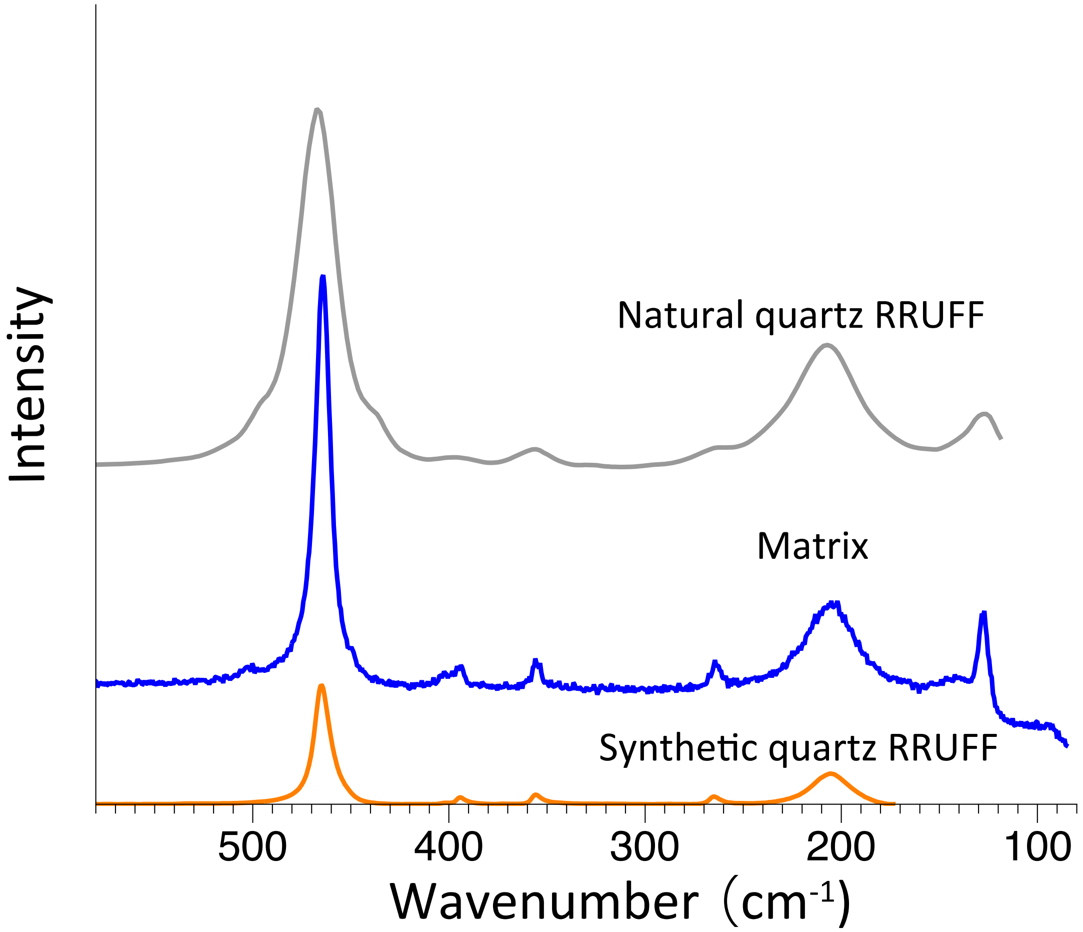

Supplementary Figure 4. Raman spectra in the 580-80 cm^{-1} region of the natural quartz, matrix and synthetic quartz RRUFF. RRUFF ID: (R040031 and R110104)

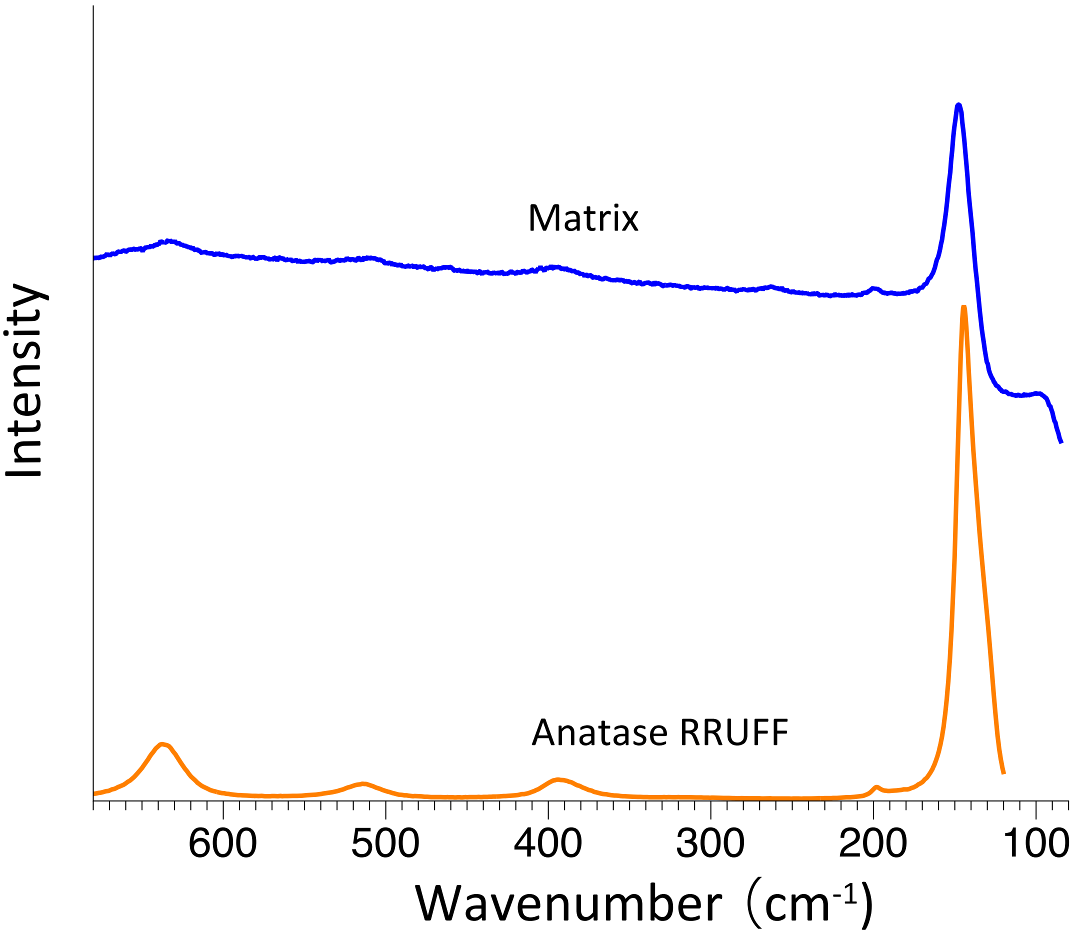

Supplementary Figure 5. Raman spectra in the 680-80 cm⁻¹ region of the matrix and anatase RRUFF. RRUFF ID: (R070582)

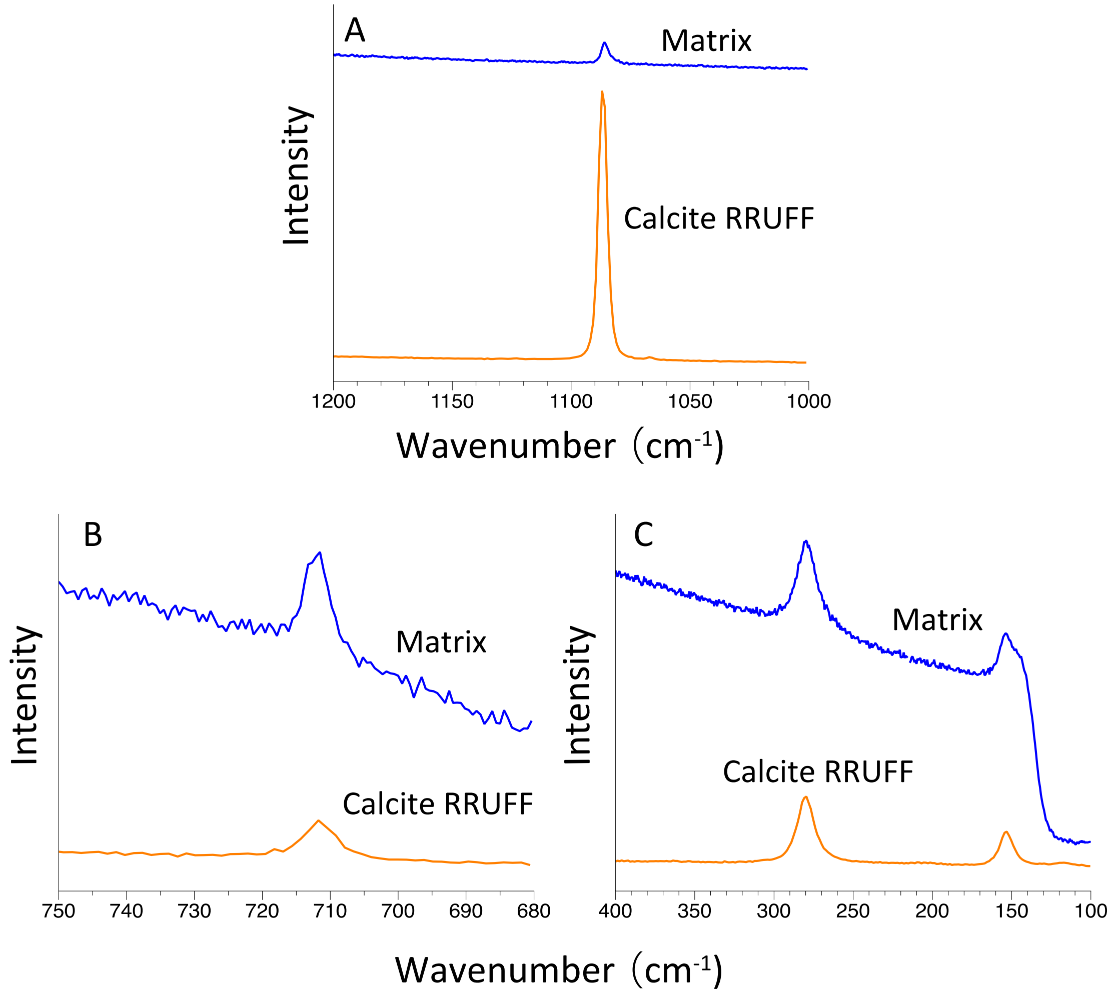

Supplementary Figure 6. (A), (B) and (C) Raman spectra in the 1200-1000 cm^{-1}, 750-680 cm^{-1} and 400-100 cm^{-1} regions of the matrix and calcite RRUFF. RRUFF ID: (R040070.3)
